# Granulomatosis with Polyangiitis Presenting as Pauci-Immune Crescentic Glomerulonephritis in Pregnancy

**DOI:** 10.1155/2016/1075659

**Published:** 2016-05-11

**Authors:** Ryan Kunjal, Raafat Makary, Andreea Poenariu

**Affiliations:** ^1^Department of Internal Medicine, University of Florida College of Medicine, Jacksonville, FL 32209, USA; ^2^Department of Pathology, University of Florida College of Medicine, Jacksonville, FL 32209, USA; ^3^Department of Nephrology, University of Florida College of Medicine, Jacksonville, FL 32209, USA

## Abstract

Antineutrophil cytoplasmic antibody (ANCA) associated vasculitis rarely affects females of reproductive age. A 28-year-old African American woman presented at 8 weeks of gestation with intractable vomiting attributed to hyperemesis gravidarum. She was found to have acute kidney injury that was unresponsive to vigorous fluid resuscitation and urine sediment examination was suggestive of an underlying glomerulonephritis. Serum c-ANCA and PR3 were elevated and there was no peripheral eosinophilia. During her course she also developed one episode of small volume hemoptysis with right upper lobe infiltrates on CT Chest. There were no cutaneous manifestations of vasculitis or upper respiratory symptoms. Renal biopsy revealed a pauci-immune crescentic glomerulonephritis (PICGN). The diagnosis was consistent with granulomatosis with polyangiitis (GPA). Management initially comprised teratogen sparing agents; steroids, intravenous immunoglobulin; and plasma exchange. The response was suboptimal and she became dependent on daily renal replacement therapy. Ultimately the pregnancy was terminated allowing for traditional treatment approaches with dramatic effect. This is the first case of GPA presenting as PICGN in pregnancy and highlights the challenges of its management.

## 1. Introduction

Antineutrophil cytoplasmic antibody (ANCA) associated vasculitis (AAV) consists of a group of small vessel vasculitides that often causes pauci-immune crescentic glomerulonephritis (PICGN). This group includes microscopic polyangiitis (MPA), granulomatosis with polyangiitis (GPA, formerly called Wegener's granulomatosis), eosinophilic granulomatosis with polyangiitis (EGPA, formerly Churg-Strauss syndrome), drug induced vasculitis, and a fifth type called “renal limited vasculitis” (RLV) [1]. Whilst it occurs across all age groups, it is most frequent in males in their 5th to 7th decades and only rarely affects young females of reproductive age [2]. There have only been a few cases reported of GPA occurring de novo in pregnancy and, to our knowledge, the following is the first reported case of GPA presenting as PICGN in a pregnant female.

## 2. Case

A 28-year-old African American female presented to the emergency department with nausea and intractable vomiting of 2-week duration. She had no urinary complaints and denied fever, diarrhea, and abdominal pain. There was no history of asthma or autoimmune illnesses. At triage she reported a last menstrual period 8 weeks priorly and her urine pregnancy test was positive. She was afebrile, tachycardic at 120/min, and normotensive at 130/83 mmHg and her physical examination was remarkable only for dry mucus membranes. Initial laboratory investigations revealed renal impairment with BUN 8.57 mmol/L (24 mg/dL), creatinine 385.4 *μ*mol/L (4.36 mg/dL), and estimated GFR 14.6 mL/min/1.73 m^2^ (MDRD study equation). She had a normocytic anemia with hemoglobin 9.5 g/dL and MCV 80 fl but no leucocytosis or eosinophilia. Urinalysis revealed 38 WBC/hpf, 478 RBC/hpf, and 300 mg/dL protein. A clean catch urine culture was negative for bacterial growth. Ultrasonography of kidneys was essentially normal and confirmed a single live intrauterine gestation.

**Figure 1:**
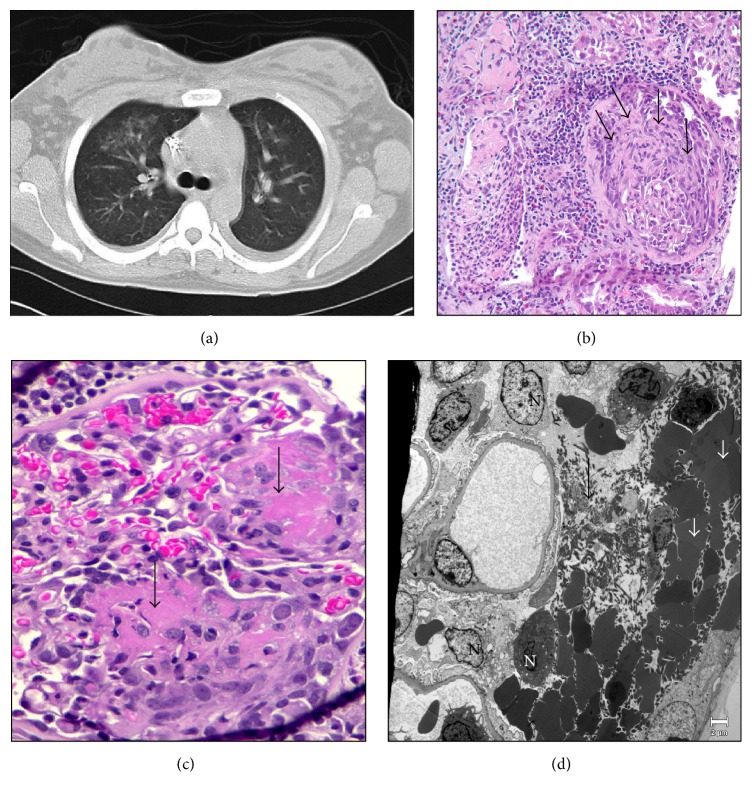
(a) CT scan of the chest done to investigate hemoptysis showing right upper lobe infiltrates. (b) Large cellular crescent distending Bowman's space and compressing the glomerular tuft (arrow), interstitial chronic inflammation, and tubular epithelial reactive/regenerative changes (HE stain ×20). (c) Necrotizing lesion (fibrinoid necrosis) in the glomerular tuft (arrow) with red blood cells (HE stain ×40). (d) EM-Fibrin tactoids (long arrow), dysmorphic red blood cells (short arrow), and inflammatory cells (N) in Bowman's space. No immune type electron dense deposits in glomerular capillary basement membrane (EM ×2900).

She was initially admitted for further management of presumed prerenal acute kidney injury as a consequence of hyperemesis gravidarum. She was vigorously resuscitated with isotonic intravenous fluids and her nausea was controlled with antiemetics. Despite these efforts, her renal function continued to worsen and urine sediment revealed several dysmorphic red blood cells. The urine spot protein to creatinine ratio was 2.0. During this time, she also developed one episode of small volume hemoptysis with right upper lobe infiltrates on CT Chest as shown in Figure 1(a). She had no upper respiratory tract complaints. Laboratory tests were done to further evaluate the underlying glomerulonephritis. C3, C4, HIV, viral hepatitis panel, serum protein electrophoresis, ANA, and dsDNA were all negative. However, positive c-ANCA (titer, 1 : 640) and PR3 > 100 *μ*/mL (normal range: 0–3.5 *μ*/mL) were noted. Anti-MPO Ab, p-ANCA, and anti-GBM were negative. A left renal biopsy was done which showed pauci-immune necrotizing glomerulonephritis with crescents in about 50% of glomeruli as shown in Figures 1(b), 1(c), and 1(d). There was also acute tubular injury with regenerative epithelial changes and red blood cells in the tubular lumen. There were no deposits of IgG, IgA, IgM, C3, or C1q on immunofluorescence. In light of these biopsy findings, the clinical features combined with elevated c-ANCA and PR3, a diagnosis of GPA was made. Her Birmingham Vasculitis Activity Score (BVAS) was 22.

Her clinical condition continued to deteriorate as she developed respiratory distress from pulmonary edema and bilateral pleural effusions secondary to acute kidney injury. Her creatinine peaked at 755.8 *μ*mol/L (8.55 mg/dL) and she required daily intermittent hemodialysis (IHD). Given the paucity of previous studies and in an attempt to avoid use of teratogenic immunosuppressants, individualized therapy was initiated with pulse steroids, using 500 mg of methylprednisolone for 3 doses and intravenous immunoglobulin (IVIG) at 400 mg/kg for 5 days. She was then started on 1 mg/kg of oral prednisone. Her renal indices did not demonstrate any improvement and plasma exchange (PLEX) was done for seven treatments. This too was unsuccessful and after lengthy discussions and counseling she made the decision to terminate her pregnancy at 12 weeks of gestation. She was then started on standard induction therapy with oral cyclophosphamide (CYC) at 2 mg/kg/day which resulted in dramatic improvement and hemodialysis was no longer required. At discharge her BVAS was 8. A summary of her clinical course is outlined in Table 1.

## 3. Discussion

PICGN is a medical emergency characterized by hematuria, proteinuria, anemia, and renal failure, all of which were seen in our patient. There are 3 main categories underlying crescentic glomerulonephritis based on changes at the cellular level and immune-fixation patterns. The first of these, anti-GBM disease, has been described in 8 previous cases with occurrence de novo in pregnancy [3]. Secondly there is a group caused by immune complex deposition as can be seen in systemic lupus erythematosus and IgA nephropathy. The third are the pauci-immune diseases, with little or no immunofluorescence staining, and etiologies include those that cause AAV as outlined previously. Amongst this group, only 3 cases describing de novo MPA in pregnancy presenting as PICGN have been reported [4, 5]. This case is the first report of de novo GPA in pregnancy manifesting primarily with renal involvement as PICGN.

GPA is a systemic small vessel vasculitis of unknown etiology characterized by the presence of necrotizing granulomatous inflammation of the respiratory tract, necrotizing vasculitis of small- to medium-sized vessels, and glomerulonephritis. There have been only 42 reported cases in 33 patients of active GPA occurring in pregnancy, but most of these involved exacerbation of the underling chronic disease. Nevertheless, 16 were newly diagnosed cases and the presenting features included intracranial bleeding, acute limb ischemia, sinusitis, and pulmonary hemorrhage [6]. Our patient denied any respiratory symptoms prior to presentation; however, during her stay she did develop hemoptysis and a transient pulmonary infiltrate in keeping with GPA. Serological tests with positive c-ANCA and PR3 along with biopsy evidence ofcrescentic glomerulonephritis solidified her diagnosis.

There is limited information as to whether pregnancy affects the course of AAV or whether AAV itself affects pregnancy outcomes. Disease activity at pregnancy onset seems to be a main indicator of poor outcomes, whilst those with longstanding remission prior to conception do best [7]. In general whether pregnancy occurs in active preexisting disease or in de novo cases, the outcome is unfavorable to both mother and child, with increased complications [8]. The management of active AAV in pregnant patients is a challenge as the risks of treatment to both mother and fetus must be balanced. There is no consensus on the approach to treatment of these patients as it still remains a very rarely described entity. However, there is significant agreement between experts on the compatibility of antirheumatic drugs during pregnancy [9].

CYC is the typical first line agent for GPA; however, it carries significant teratogenic potential during the organogenetic period (<12 weeks) and can cause spontaneous miscarriages and birth defects [5]. Our patient's period of gestation fell into this critical window and thus alternative treatment modalities were sought in addition to pulse dosed steroids. High dose IVIG has been proposed as second line treatment in GPA when standard agents are contraindicated. Masterson et al. [10] first described the successful induction of remission with IVIG and systemic steroids in a woman diagnosed with de novo GPA during the first trimester of pregnancy. However, our patient responded poorly to this therapy and there are no large scale studies that evaluated its optimal dosing, efficacy, and safety in pregnant patients. The other option tried in this patient was antibody immune-adsorption via PLEX which has been demonstrated to be safe and well tolerated in pregnant women when used for other conditions [11]. Evidence thus far has shown some benefit of this therapy in AAV; however, many key questions remain unanswered [12]. Agents not tried in our patient included azathioprine (AZA) and rituximab (RTX). AZA is relatively safe in pregnancy when used in women previously diagnosed with GPA to maintain remission. Alfhaily et al. used AZA as part of their induction regimen for GPA presenting in the third trimester; however, remission was not effective and durable [13]. Our patient required robust induction therapy, so AZA was not used initially, but she has been maintained on it without relapse. RTX has also been used in severe AAV with some promising results, but data still remains limited on its use in both pregnancy and GPA [14].

This case highlights a rare occurrence of GPA presenting as PICGN in pregnancy. Ethical considerations limit the conduct of large randomized control studies in this subgroup of pregnant women to develop guidelines on management. Thus, reports of these cases remain invaluable to assist other clinicians who may be faced with similar challenges. The successful response of therapies tried in other patients was not effective in our patient. Not only was her disease aggressive, but also she presented at the most sensitive time in the development of her fetus. Unfortunately, her pregnancy was terminated to allow standard treatment approaches which ultimately led to remission of her illness.

## Figures and Tables

**Table 1:** Showing the clinical course and response to treatment.

Day after presentation	0	10	18	27	50	120
Treatment	Admission	IHD initiated	Completed pulse steroid + IVIG	Completed PLEX	Discharged off IHD for 1 week, Day 18 CYC	3 months after CYC induction
Period of gestation (weeks completed)	8	9	10	11	Postpartum	Postpartum
Ser. creatinine (*µ*mol/L)	385.4	755.8	On IHD	On IHD	291.7	176.8
Urine output (mL/24 hr)	125	75	175	100	2000	2250
Protein/creatinine ratio	2.0	10.5	—	6.4	3.1	1.0
Proteinuria (mg/dL)	300	300	300	300	100	100
Hematuria (RBC/hpf)	478	522	544	1824	80	27

## References

[1] Jennette J. C., Falk R. J., Bacon P. A. (2013). 2012 revised International Chapel Hill Consensus Conference Nomenclature of Vasculitides. *Arthritis and Rheumatism*.

[2] Rowaiye O. O., Kusztal M., Klinger M. (2015). The kidneys and ANCA-associated vasculitis: From pathogenesis to diagnosis. *Clinical Kidney Journal*.

[3] Thomson B., Joseph G., Clark W. F. (2014). Maternal, pregnancy and fetal outcomes in de novo anti-glomerular basement membrane antibody disease in pregnancy: a systematic review. *Clinical Kidney Journal*.

[4] Cetinkaya R., Odabas A. R., Gursan N. (2002). Microscopic polyangiitis in a pregnant woman. *Southern Medical Journal*.

[5] Porres-Aguilar M., Figueroa-Casas J. B., Porres-Muñoz M., Elliott C. G. (2011). A 38-year-old pregnant woman with hemoptysis and acute renal failure. *Respiration*.

[6] Devakumar V. N., Castelino M., Chow S.-C., Teh L.-S. (2010). Wegener's granulomatosis in pregnancy: a case report and review of the medical literature. *BMJ Case Reports*.

[7] Auzary C., Le Thi Huong D., Wechsler B., Vauthier-Brouzes D., Piette J.-C. (2000). Pregnancy in patients with Wegener's granulomatosis: report of five cases in three women. *Annals of the Rheumatic Diseases*.

[8] Tuin J., Sanders J. S. F., de Joode A. A. E., Stegeman C. A. (2012). Pregnancy in women diagnosed with antineutrophil cytoplasmic antibody-associated vasculitis: outcome for the mother and the child. *Arthritis Care and Research*.

[9] Götestam S. C., Hoeltzenbein M., Tincani A. (2015). The EULAR points to consider for use of antirheumatic drugs before pregnancy, and during pregnancy and lactation. *Annals of the Rheumatic Diseases*.

[10] Masterson R., Pellicano R., Bleasel K., McMahon L. P. (2004). Wegener's granulomatosis in pregnancy: a novel approach to management. *American Journal of Kidney Diseases*.

[11] Dittrich E., Schmaldienst S., Langer M., Jansen M., Hörl W. H., Derfler K. (2002). Immunoadsorption and plasma exchange in pregnancy. *Kidney and Blood Pressure Research*.

[12] Walters G. (2016). Role of therapeutic plasmapheresis in ANCA-associated vasculitis. *Pediatric Nephrology*.

[13] Alfhaily F., Watts R., Leather A. (2009). Wegener's granulomatosis occurring de novo during pregnancy. *Clinical and Experimental Rheumatology*.

[14] Lally L., Spiera R. (2016). B-cell-targeted therapy in systemic vasculitis. *Current Opinion in Rheumatology*.

